# Comparison of Acupuncture Effect on Blood Perfusion between Needling Nonacupoint on Meridian and Needling Nonacupoint off Meridian

**DOI:** 10.1155/2013/426052

**Published:** 2013-08-12

**Authors:** Wei-Bo Zhang, Ling-Ling Wang, Heng-Hui Xie, Hong Li, Yu-Ying Tian

**Affiliations:** ^1^Institute of Acupuncture & Moxibustion, China Academy of Chinese Medical Sciences, Beijing 100700, China; ^2^Liulitun Community Health Centre, Chaoyang District, Beijing 100026, China; ^3^Beijing Chaoyang Hospital, Beijing 100020, China

## Abstract

To verify the ancient theory of rather missing the acupoint than missing the meridian, acupuncture at nonacupoint on meridian and acupuncture at nonacupoint off meridian were performed, respectively. The blood perfusion (BP) on the calf around bladder meridian area was measured with a laser Doppler perfusion imager before, during, and after acupuncture. The whole scanning field was divided into seven subareas, and mean BP on each area was calculated. The ratio of mean BP between a subarea and a reference subarea was gotten, and then the change rate was calculated as ratio change rate (RCR). The results showed that RCR on bladder meridian area and around Chengshan (BL57) during or after acupuncture at nonacupoint on meridian was significantly higher than that at nonacupoint off meridian, which supports the ancient theory. Such differences may be attributable to some factors that can facilitate the signals transmission and produce a better acupuncture effect, such as richer nerve terminals, blood vessels, and mast cells which can produce stronger signals on the acupoints and the low hydraulic resistance channel along meridians which plays a role of signal transmitting channel to get a better effect of acupuncture.

## 1. Introduction

The ancient theory of “rather missing the acupoint than missing the meridian” is an important principle to guide the acupuncture clinic originated from Yang's “Compendium of Acupuncture and Moxibustion” in *Ming *dynasty [1]. In locating acupoints, patient's fingers are often used as a ruler, and *cun* serves as unit of length for measurement which is called finger *cun*. For instance, the length between the two medial ends of the twisted folds of the middle finger when bent, or the width of the phalangeal joint of the thumb is taken as 1 cun, and the maximal width of the four fingers (viz., the first finger, middle finger, ring finger, and little finger) held together with the hand open is taken as a unit of measurement of 3 cun. But in acupuncture clinic, locating of acupoints with patient's finger is not convenient and often replaced by acupuncturist's finger which is easy to miss the right position as the *cun* from the acupuncturist is often different from the “*cun*” from the patient. In addition, locating of the acupoint on meridian is also conducted by anatomic signs or by the patient's feeling when pressing the specific place on body surface with a finger. Thus, it is possible to miss acupoints or miss meridians during the locating. The ancient theory of “rather missing the acupoint than missing the meridian” implies a possibility that the effect of acupuncture at the nonacupoint on the meridian is superior to that of acupuncture at nonacupoint off the meridian. 

Acupuncture effect was represented partly by blood perfusion (BP) or blood flow (BF) in several experiments [2–7]. The imaging technique which can measure BP level on skin has been used in evaluating the efficiency of acupuncture. It has great advantages of being objective and having ability of measuring BP level on different parts of body surface. By this technique, we have observed the effect of acupuncture at Hegu (LI 4) and found three different effects such as local effect, holistic effect, and meridian effect which is important to understanding of the relationship between acupuncture and meridians [4]. This study was undertaken to compare the effects of acupuncture at nonacupoint on the meridian and acupuncture at nonacupoint off the meridian to examine the ancient theory of “rather missing the acupoint than missing the meridian.” 

## 2. Materials and Methods

### 2.1. Subjects

20 healthy volunteers were from China Academy of Chinese Medical Sciences and Beijing University of Chinese Medicine. Of them, 11 were male and 9 female, ranging in age from 19 to 55 years. They had no illnesses or obvious discomfort within a week and had not taken medicine in the past one month before the test. All the participants had an adequate understanding of the procedure and purpose of this trial. 

### 2.2. Instruments and Environment

The BP on the skin of calf around bladder meridian area was measured by a PeriScan PIM II laser Doppler perfusion imager (made in Sweden). The instrument was set to medium resolution and 50 × 64 (3200) points. The scan area was about 15 × 20 cm and each scan took about 3 minutes. The acupuncture needle of 0.30 × 30 mm (Huan Qiu, Suzhou, China) was used in this test. The test was carried out in a dark room. The room temperature was kept between 26 and 28°C. The fluctuation in the temperature was less than ±1°C during one experiment.

### 2.3. Experimental Procedure

Each subject received twice measurements with an interval of more than two days between the two measurements and chose to acupuncture at nonacupoint on the meridian first or at nonacupoint off the meridian first at random. Before the measurement, subject was asked to lie on bed for ten minutes to adapt to the room temperature. Measuring of BP before acupuncture was given for one time and then the acupuncture was started.

**Figure 1:**
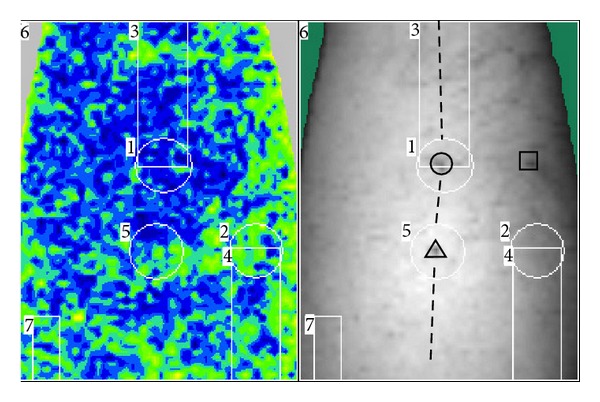
The position of acupunctured points and the area divisions for analyzing mean blood perfusion. ○ denotes Chengshan (BL57), and the broken line denotes bladder meridian. △ denotes the nonacupoint on bladder meridian, and □ denotes the nonacupoint off bladder meridian. The upper set of the figure is the direction of foot, and the inferior set is the direction of head.

**Figure 2:**
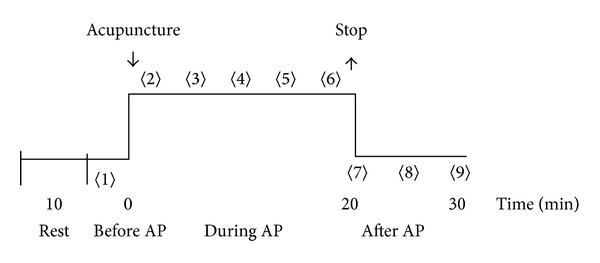
The procedure of the experiment. The number in 〈〉 represents the scanning sequence for measurement of blood perfusion, and AP denotes acupuncture.

Acupuncture was performed at two selected points. One point was located on 2 cm above Chengshan (BL57), on Bladder Meridian of Foot Taiyang, as nonacupoint on the meridian, and the other point on Chengshan (BL 57) level, 2 cm lateral to Bladder Meridian of Foot Taiyang, as nonacupoint off the meridian (Figure 1). One of the two points was acupunctured first. The needle was inserted into 0.5 to 1.0 cun depth that is the standard depth of Chengshan (BL 57), and uniform reinforcing-reducing manipulation was applied for about one minute to induce needling reaction as much as possible. After obtainment of needling reaction, the needle was remained. During the needle retention period, the BP was continuously measured for five times, which took totally about 20 minutes. And then, the needle was withdrawn. After withdrawal of the needle, the BP was measured again for three times, which took about 10 minutes. The procedure is shown in Figure 2. 

### 2.4. Calculation of Mean BP and Statistics

The whole measured area on the calf was divided into seven subareas (Figure 1). Area 1 was Chengshan (BL 57). Area 2 was a control point at comparative position of Chengshan (BL 57) when giving acupuncture at nonacupoint off meridian. Area 3 was the meridian area. Area 4 was a control area comparative to area 3. Area 5 was the area around the needling points. It was on bladder meridian (around △ in Figure 1) in nonacupoint on meridian group and lateral to bladder meridian in nonacupoint off meridian group (around □ which has not been shown in Figure 1). Area 6 was the whole measured area. Area 7 was a reference area that has no obvious relation with acupuncture and meridian.

After the seven subareas were divided, mean BP level in each area was calculated automatically by LDPIwin2.5 software which was obtained with the instrument. An important data analysis was achieved in our previous study that the BP at a reference area was gotten to represent the whole body change which was influenced by various unknown factors and by nonspecific effect of acupuncture [4, 8]. The specific local and meridian effects of acupuncture were overlapped on the whole body change. To enlarge the specific effects and minimize the fluctuation and nonspecific whole body effect of acupuncture, the ratio (BP_*i*_/BP_rf_) between the BP on one area (BP_*i*_) except the reference area (area 7) and the BP on reference area (BP_rf_) was calculated as RBP_*i*_ (*i* is 1 to 6), and then the change rate of the ratio by time was calculated as (RBP_*t*_ − RBP_*b*_)/RBP_*b*_, (*b* denotes before acupuncture) again on each area (area 1 to area 6) which was called ratio change rate (RCR) in percentage (*t* is 2 to 9, representing nine measured times). Paired two-tailed *t*-test was used to compare the difference of RCR between nonacupoint on meridian group (on meridian group) and nonacupoint off meridian group (off meridian group). *P* < 0.05 was set as the significant difference.

## 3. Results

After the ratio between the BP on one area and the BP on reference area (RBP_*i*_) was calculated and ratio change rate (RCR_*t*_) at different times was calculated, the mean RCRs of 20 subjects at six subareas are shown in Table 1. 

**Figure 3:**
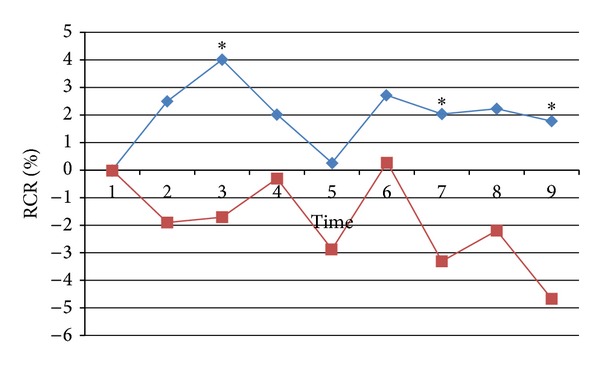
Ratio change rate (RCR) of BP on bladder meridian (area 3) when needling at nonacupoint on bladder meridian (blue line) and at nonacupoint off bladder meridian (red line). * denoting a significant difference (*P* < 0.05) between the two groups.

From the Table 1, the RCR at area 1, Chengshan (BL 57) and the control area (area 2) had no significant difference between needling the two points. However, the RCR on bladder meridian (area 3) in on meridian group was significantly higher than that in off meridian group during the needle retention period (time 3, *P* < 0.05) and after withdrawal of the needle (time 7, *P* < 0.05 and time 9, *P* < 0.01, see Figure 3), which implied a stronger effect along bladder meridian when acupuncturing at nonacupoint on the meridian. The average RCR during the whole observed periods was 2.2% in on meridian group and −2.1% in off meridian group, with total 4.3% difference between the two groups.

The difference in the RCR of BP on control area (area 4) that is similar to the size of meridian area (area 3) was not significant between on meridian group and off meridian group during most periods. However, at time 9 after withdrawal of the needle, the difference became significant, which indicates that acupuncture at nonacupoint on the meridian possesses a stronger lasting effect. 

**Figure 4:**
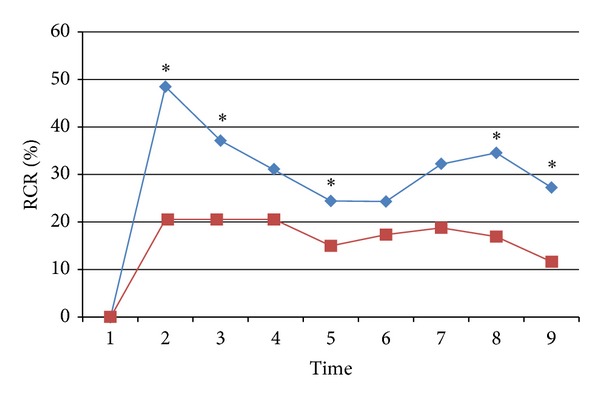
The local effect of ratio change rate (RCR) of BP around the needling points. Blue line denotes RCR in acupuncture at nonacupoint on the meridian, and red line denotes RCR in acupuncture at nonacupoint off the meridian. * denoting a significant difference (*P* < 0.05) between the two groups.

The local effect around needling points was compared between acupuncture at the two nonacupoints (Figure 4). RCR in acupuncture at nonacupoint on the meridian was significantly higher than that in acupuncture at nonacupoint off the meridian during needle retention period (time 2, *P* < 0.01; time 3, *P* < 0.01; time 5, *P* < 0.05) and after withdrawal of the needle (time 8, *P* < 0.05; time 9, *P* < 0.05).

**Figure 5:**
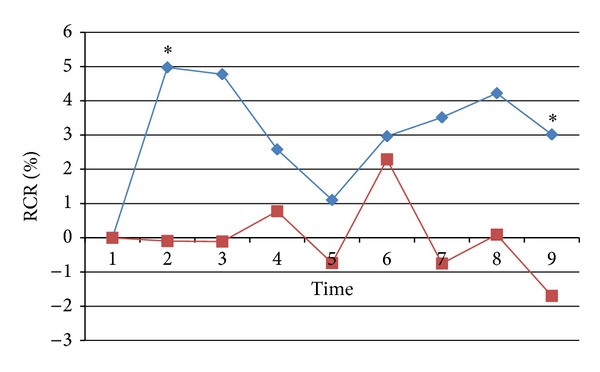
Ratio change rates of BP in on meridian group (blue line) and off meridian group (red line). * denoting a significant difference between the two groups.

The holistic effect (area 6) still existed in on meridian group even when the pure holistic effect without local effect and meridian effect was diminished by calculation of the ratio, while it was nearly diminished (around zero) in off meridian group (Figure 5).

## 4. Discussion

From the result of the previous experiment, a stronger effect of acupuncture on blood perfusion was found when needling the nonacupoint on meridian than needling the nonacupoint off meridian. 

### 4.1. The Methodology of Studying Meridian

Many studies focused on comparing the difference of acupuncture effects between needling acupoint on meridians, and nonacupoint off meridians while little is known about the difference between needling nonacupoint on meridian and nonacupoint off meridian. Zheng et al. measured the blood perfusion at deep tissue using a needle-type detector and found little difference in increase of BP during acupuncture [9]. This result was similar to our observation that the difference in BP level on area 1 Chengshan (BL 57) which is a point-like small area was not significant between needling the two nonacupoints. The reason for this may be that the detected area on the meridian is too small to collect enough changes. When the detected area was enlarged on more meridian region like the field of rectangle in area 3, sufficient change could be obtained from the meridian to show the difference between meridian and bilateral control areas. So, an imaging technique is important in acupuncture and meridian study. Diminishing fluctuation from the whole body is also important as there is an overlapping from unavoidable environmental and mental noises on the real changes induced by acupuncture through meridians. The technique of getting fluctuation from a reference area and calculating the ratio with other areas proved to be effective in our previous studies [4, 8] and this study, which could be regarded as a technique of improving signal-to-noise ratio on meridians and acupoints.

### 4.2. The Coherent Function of Meridians

It remains a question that what is the role of meridian in acupuncture clinic. The ancient theory told us that you will make a mistake if you do not know the meridians. But modern acupuncture pays little attention to the importance of meridians. The precision of selecting an acupoint during acupuncture is an important factor to influence the acupuncture effect. In Song dynasty, a doctor must pass an examination of selecting acupoints on a bronze acupuncture figure in that water can flow out if he needles an acupoint correctly. The precision of selecting an acupoint depends on acupuncturist's experience at present during which the position of a selected point may miss the real acupoint but still on the meridian line or miss the real acupoint and miss the meridian at the same time. Acupoints along fourteen meridians are the main points used to apply acupuncture. Many acupoints along one meridian have similar function in treatment of the disease. The mechanism of the fact was speculated by ancient doctors that a “*qi* river (channel)” passes across these acupoints to transport the signals and effects. If the speculation is correct, it is reasonable to deduce that nonacupoints, but still locating on meridians can facilitate the transportation and therefore have a better effect. 

Zhao et al. found an increase in gastric electric activity when acupuncturing Sibai (ST2) and Dichang (ST4) along the stomach meridian; however, no obvious changes were found when acupuncturing a nonmeridian point beside Sibai (ST2) and a point on small intestine meridian [10]. Huang et al. observed the blood flow on basilar artery when acupuncturing 5 acupoints, 4 nonacupoints along bladder meridian, and 9 nonmeridian points located on 2 cm lateral to the 9 points on the meridian. They found that the effect was the highest in the acupoints, secondary in nonacupoints, and the lowest in the nonmeridian points, which had shown an important role of acupoints and meridians [11]. But they did not quantitatively compare the difference between needling nonacupoint and nonmeridian point with the same departure from an acupoint. 

**Figure 6:**
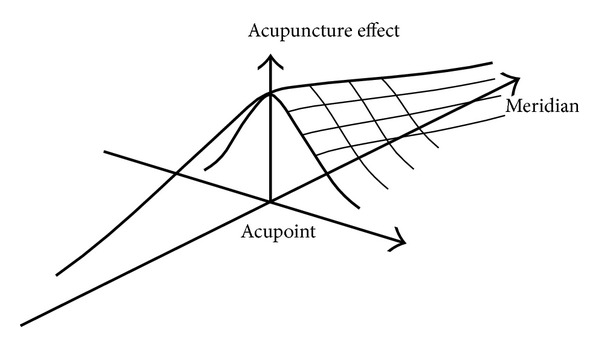
An illustration of acupuncture effect related with acupoints and meridians. There is a descending of effect from acupoint both along meridian and perpendicular to the meridian while the falling gradient is smooth along the meridian than perpendicular to the meridian.

Our new result showed that when the points have the same departure from the acupoint, the local effect and meridian effect in nonacupoint are significantly higher than that in nonmeridian point. To see the local effect around the needling point immediately after acupuncture and combined with the data obtained before, 60.1%, 48.5%, and 20.5% increase in turn in acupoint, nonacupoint on meridians, and nonacupoint off meridians, which further proved the rule of acupuncture effect related to acupoints and meridians. The rule has been described roughly in Zhang's book “what is the meridian” in 1997 [12] and is redrawn in Figure 6 more vividly. The ancient theory of “rather missing acupoints than missing meridians” got a scientific elucidation. 

### 4.3. The Mechanism of Better Effects in Acupoints and Meridians

The mechanism of the strongest acupuncture effect of needling acupoints might be due to the dense distributions of nerve terminals, blood vessels, and mast cells at the acupoint sites [13–15] at which stronger acupuncture signals like substance P (SP) and histamine can be produced by the needling through axon reflex [16]. The existed meridian channels can facilitate the process through transporting SP from nerve terminals to mast cells and transporting SP and histamine released from mast cells to the blood vessel to enhance the blood perfusion around the needling site and along the meridian as all the acupoints are located on the meridians.

The stronger effect of acupuncture at nonacupoints on meridians can be attributed to the existence of low hydraulic resistance channel along meridian which was found by Zhang et al. [17]. The channel allows more interstitial fluid flow and causes a transfer of histamine, SP, and other chemicals along meridians as a kind of volume transmission in peripheral tissue [18]. The transfer of chemicals and a series of axon reflexes along meridians can produce a propagated sensation along meridians [19] which is a famous phenomenon in meridian study. The transmission of chemical and neural signals along meridians will also cause the acupuncture effect along meridians [4] and act on the organs where the meridian passes across. As long as the needle is inserted on a meridian, the signal is easy to be transported by the channel and easy to get an acupuncture effect, thus benefiting the treatment. So, it is important to keep the acupuncture on meridians. The ancient theory of “rather missing the acupoint than missing the meridian” has gotten an experimental verification and scientific understanding. 

## 5. Conclusion

Nerve terminals, blood vessels, and mast cells can be regarded as the substance of acupoints through which stronger acupuncture signals can be produced to get the best effect. The low hydraulic resistance channel of interstitial fluid represents the entity of meridian channels which plays a role of signal transmitting channel to get a better effect of acupuncture. 

## Figures and Tables

**Table 1:** Ratio change rate of blood perfusion on difference areas during various periods of acupuncture (Mean ± SD, *n* = 20).

	Area 1	Area 2	Area 3	Area 4	Area 5	Area 6
On meridian						
Time 2	5.12 ± 11.48	7.0 ± 12.56	2.50 ± 8.69	2.56 ± 6.07	48.5 ± 37.09**	4.98 ± 7.24*
Time 3	6.26 ± 11.33	4.5 ± 14.02	4.01 ± 9.80*	2.89 ± 9.76	37.1 ± 25.84**	4.78 ± 7.74
Time 4	3.71 ± 9.69	2.6 ± 12.95	2.02 ± 6.08	0.55 ± 7.24	31.0 ± 22.44	2.58 ± 6.29
Time 5	2.55 ± 10.65	1.0 ± 11.86	0.26 ± 7.63	0.46 ± 7.26	24.4 ± 20.06*	1.10 ± 7.41
Time 6	1.74 ± 10.14	3.5 ± 12.46	2.7 ± 8.57	1.82 ± 7.06	24.3 ± 16.63	2.97 ± 6.84
Time 7	1.93 ± 11.22	2.1 ± 14.46	2.0 ± 7.62*	2.00 ± 8.32	32.0 ± 26.96	3.52 ± 7.77
Time 8	4.03 ± 13.67	4.1 ± 12.86	2.2 ± 10.41	2.68 ± 9.30	34.5 ± 30.77*	4.22 ± 8.75
Time 9	1.75 ± 9.38	3.1 ± 11.91	1.7 ± 7.67**	2.34 ± 6.63*	27.2 ± 25.12*	3.02 ± 6.16*
Off meridian						
Time 2	1.11 ± 9.89	3.89 ± 10.13	−1.90 ± 5.45	−1.0 ± 7.70	20.5 ± 15.10	−0.10 ± 6.08
Time 3	−0.32 ± 9.50	2.89 ± 11.40	−1.70 ± 5.09	−0.8 ± 8.00	20.2 ± 13.13	−0.11 ± 5.50
Time 4	1.96 ± 10.96	1.52 ± 7.66	−0.31 ± 7.90	0.47 ± 6.23	20.4 ± 12.56	0.77 ± 5.72
Time 5	−2.09 ± 9.65	1.74 ± 9.63	−2.88 ± 4.48	−1.2 ± 7.50	14.9 ± 8.21	−0.74 ± 5.27
Time 6	1.12 ± 9.69	5.68 ± 12.57	0.27 ± 5.69	0.91 ± 8.87	17.3 ± 11.45	2.29 ± 5.56
Time 7	−2.53 ± 12.25	2.96 ± 9.57	−3.31 ± 8.74	0.37 ± 7.80	18.7 ± 15.58	−0.75 ± 6.43
Time 8	−0.01 ± 10.27	2.54 ± 9.09	−2.20 ± 4.60	−0.1 ± 8.04	16.9 ± 14.14	0.09 ± 5.46
Time 9	−3.75 ± 9.78	−0.3 ± 9.68	−4.67 ± 7.50	−1.8 ± 7.04	11.6 ± 12.05	−1.70 ± 5.05

*P < 0.05, **P < 0.01 as compared between on meridian group and off meridian group at same time period on the same area.
